# Adaption and Cultural Validation of the Quality in Psychiatric Care-Outpatient Staff (QPC-OPS) Instrument to a Norwegian Community Mental Health Context

**DOI:** 10.1007/s11414-022-09788-2

**Published:** 2022-06-15

**Authors:** Hege Skundberg-Kletthagen, Agneta Schröder, Lars-Olov Lundqvist, Øyfrid Larsen Moen, Marianne Thorsen Gonzalez

**Affiliations:** 1 Faculty of Medicine and Health, Institute of Health Sciences, Norwegian University of Science and Technology (NTNU), P.O box 191, N-2802 Gjøvik, Norway; 2 Faculty of Medicine and Health, University Health Care Research Center, Örebro University, Örebro, Sweden; 3 Faculty of Health and Social Sciences, Institute of Nursing and Health Sciences, University of South-Eastern Norway (USN), Notodden, Norway

## Abstract

The aim was to culturally adapt and validate the Swedish Quality in Psychiatric Care-Outpatient Staff (QPC-OPS) instrument for use in a Norwegian community mental health service context. The translated and culturally adapted instrument was named Quality in Psychiatric Care-Community Outpatient Staff (QPC-COPS). Three expert panels of mental health staff (*n* = 9) assessed the face and content validity. The internal consistency and test–retest reliability were assessed on a sample of community mental health staff (*n* = 64). The QCP-COPS had adequate face and content validity, and the full instrument showed excellent internal consistency (alpha = 0.90) and test–retest reliability (ICC = 0.87:0.94). In conclusion, the QPC-COPS is a valid and reliable instrument suitable for measuring staff’s perception of the quality of care they deliver in community mental health services.

## Introduction

Receiving high-quality care is the right of all patients, and it is the responsibility of all health care staff to provide this.^1^ In Norway, the provision of mental health services has over time changed from inpatient care to outpatient care, where the community mental health service has increasingly become the main provider of mental health care in municipalities. Thus, the content and quality of the community mental health service are vital to the patient’s and their family’s experiences of the quality of care.^2,3^ To ensure that care is of high quality and fulfills the formal requirements, continuous assessment and improvement are essential.^4^

Staff describe quality in care both as a multidimensional concept in the mental health care services^5^ and as a positive and normative concept, i.e., high quality in care.^6^ Systematic use of robust and validated instruments may help identify areas or scope in the delivery of care where quality improvements of mental health services are at stake.^7^ Therefore, instruments that have an explicit conceptualization of quality in care^8^ and are designed for that specific service and context ought to be developed and applied.^9^ Consequently, these instruments should also be psychometrically evaluated.^10^ One such instrument is Quality in Psychiatric Care (QPC). The QPC instrument is in line with an ambitious quality in care definition originally developed from the patient’s perspective.^11^ The QPC is a family of instruments in which core context–dependent modules are outlined for use in six different mental health contexts: psychiatric outpatient (QPC-OP)^12^, psychiatric inpatient (QPC-IP)^13^, psychiatric forensic inpatient (QPC-FIP)^14^, addiction outpatient care (QPC-AOP)^15^, community-based daily activities for people with psychiatric disabilities (QPC-DA)^16^, and housing support for people with psychiatric disabilities (QPC-H).^17^ There is also a staff version of each QPC version, but so far only the version for psychiatric forensic inpatient care (QPC-FIPS) has been evaluated.^14^ Cultural adaption of the QPC-IP and QPC-IPS has been carried out in Indonesia^18,19^ and in Spain^20^, while in Denmark the QPC-FIP and QPC-FIPS^21,22^ have been culturally adapted. Moreover, the patient-perceived quality of care in psychiatric hospitals (QPC-IP)^23^ has been adapted in Taiwan. Over the years, quality in care has undergone continuous improvements in mental health services in the Western world.^24^ Nevertheless, so far research addressing quality in care in the community mental health services is scarce, and few standardized and validated instruments for quality assessment exist. In particular, there is a need for instruments from the staff’s perspective.^25^ Gaining a better understanding of the staff’s perspective of their delivered quality in care may help identify domains where quality improvements are needed, resulting in new strategies to improve the quality of mental health services.^26^ We still know little about how staff perceive the quality in care they provide, and it would be advantageous if they were well informed on this issue.^27^ Thus, mental health staff’s ratings of the quality in care they provide may add valuable information to patients’ quality of care ratings. To accurately assess and monitor staff perception of quality in care, valid and reliable instruments are required.

This study presents the process and outcome of culturally adapting and validating the Swedish Quality in Psychiatric Care-Outpatient Staff (QPC-OPS)^12^ instrument for use in the Norwegian community mental health services. The Norwegian instrument was given the name the Quality in Psychiatric Care-Community Outpatient Staff (QPC-COPS).

### Research Objectives

The objectives of the current study were threefold:

1. To translate the Swedish QPC-OPS to Norwegian and culturally adapt the instrument to the Norwegian community mental health services.
2. To assess face and content validity of the Norwegian Quality in Psychiatric Care-Community Outpatient Staff (QPC-COPS).
3. To assess the internal consistency and stability of the QPC-COPS instrument.

## Methods

To achieve the research objectives, this study followed the process of translation and adaptation of the instruments.^28^ The overall study was carried out stepwise and involved different approaches in each of the three steps. The stepwise approaches will be presented chronologically in the following.

Mental health care services in Norway consist of community and specialist health services. The municipalities, both urban and rural, are responsible for providing community mental health care services, nursing, and care services in addition to social services and preventive efforts.^29^.

### The Swedish QPC-OPS Instrument

The Swedish instrument Quality of Care-Outpatient staff (QPC-OPS), originally developed by Schröder and Lundqvist^12^, consists of 30 items addressing eight quality in care dimensions: encounter (6 items), participation-empowerment (3 items) participation-information (5 items), discharge (3 items), support (4 items), environment (3 items), next of kin (2 items), and accessibility (4 items). All items in the QPC-OPS are related to the phrase: “I experienced that…” and responses are made on a 4-point Likert-type scale with a rating from 1 (totally disagree) to 4 (totally agree). The QPC-OPS contains background questions that address demographical and general clinical information.

To prepare the QPC-OPS instrument for use in a Norwegian context, accurate translation and cultural adaption are required.^30^

#### Step I: Translation of QPC-OPS and Cultural Adaption

The translation of the QPC-OPS instrument from Swedish to Norwegian was carried out in line with Beaton, Bombardier et al.’s^30^ descriptions of translation and back-translation. First, the QPC-OPS instrument was translated from Swedish to Norwegian by a Norwegian native speaker and an authorized translator with Swedish as a second language. Thereafter, an independent authorized translator back-translated the Norwegian version into Swedish. Second, the back-translation was discussed by the Swedish researchers (A.S. and L-O.L), and the Norwegian version was compared with the Swedish version to identify any difficulties in the translation process. Some minor corrections of the translation were made after discussions with both translators. Thereafter, a preliminary version of the Norwegian QPC-OPS was designed. Thirdly, the Swedish (A.S. and L-O.L) and the Norwegian researchers (H.S-K, Ø.L.M & M.T.G.) discussed the preliminary Norwegian version in detail and compared it with the Swedish version. Some minor differences were identified, and changes were made accordingly.

After having translated the QPC-OPS, we aimed for a cultural adaption. Due to the organizational and therefore also cultural and conceptual differences between Swedish mental health out-patient services and Norwegian community mental health services, some items in the QPC-OPS were reworded and some concepts were replaced with concepts that were more suitable and applicable for the community mental health services in Norway.

#### Step II: Assessment of Face and Content Validity

To assess the face and content validity^31^ of the QPC-COPS, we established three expert panels. Each expert panel consisted of three experienced mental health staff employed in different Norwegian community mental health service settings. Consequently, they represented three different cultures in community mental healthcare. The nine professionals were all mental health nurses or social workers with further education within mental health. They varied in age from 28 to 66 years (median 45 years), and their work experience varied from 1 to 20 years (median 6 years) in the community mental health service setting where they currently worked. See Table 1.

**Table 1:** Background characteristic of the expert panel and the participants of the test–retest

	Expert panel	Test–retest participants
*n*	9	64
Gender *n* (%)		
Male	0	3 (6)
Female	9 (100)	60 (94)
Age Md (range)	45 (28–66)	47 (24–68)
The number of years in the current community mental health service Md (range)	6 (1–20)	7 (1–34)
Occupation (*n*)		
Nurse	5	33
Social worker	4	9
Social educator		17
Occupational therapist		2
Psychologist		1
Milieu staff		1

The procedure involved the initial distribution of the QPC-COPS instrument to the expert panels. Thereafter the expert panels met two of the five researchers (H.S-K. and M.T.G.) for interviews/panel discussions at their workplace. The researchers invited the participants to assess the information provided before filling in the instrument. The face validity was addressed by asking the professionals how they perceived the QPC-COPS items in terms of the wording and whether the items fitted the specific dimension they referred to (i.e., encounter, participation, discharge, support, environment, next of kin, or accessibility). One of the researchers acted as a moderator by asking questions, while the other observed and took notes, ensuring that all relevant information from the participants was taken into consideration.^31^

The expert panels also assessed the content validity by giving written feedback on how they assessed the relevance or importance of each item in the QPC-COPS. The responses on the 30 items included three value options: 1 (less important), 2 (important), and 3 (very important). The degree to which they assessed the grade of comprehensibility was assessed on a scale from 1 (easily to understand/clear), 2 (acceptable), and 3 (hard to understand/unclear). The three response options were used in the subsequent assessment of the instrument. Regarding their perception of how understandable they deemed the items to be, items 10, 13, and 19 (see Table 3) were considered unclear. On account of these results, the three items were further clarified. In aiming for content validity, the expert panels also shared their reflections on whether and how the items fitted and reflected relevance to the Norwegian community mental health care service in which they were working.

#### Step III: Assessment of Internal Consistency and Test–Retest Reliability

To assess the internal consistency and test–retest reliability of QPC-COPS, a sample of mental health staff employed in community mental health services in 18 Norwegian municipalities were recruited by contacting line managers in the municipalities by telephone, followed by an e-mail with attached written information for distribution to potential participants. The line manager orally informed those who met the inclusion criteria of having a least 1 year of work experience in the community mental health service and then forwarded the written study information to them. In total, 64 mental health staff agreed to participate and returned a signed consent form to the first author. All of them filled out and returned the QPC-COPS instrument on two occasions with 2 weeks in between. Demographic information on the study group is given in Table 1.

### Data Analysis

The statistical software package SPSS version 22 (IBM Corp., Armonk, NY, USA) was used.

A descriptive statistical analysis of QPC-COPS was made for the first test in the test–retest. In the case of missing items (> 30%) in the different eight dimensions, imputation was performed by replacing missing data points with the participant’s mean item value of the total mean score, i.e., the case mean substitution technique.^32^ Imputation was performed on eight of the 64 questionnaires.

Internal consistency of the QPC-COPS’s total scale and dimensions was assessed with Cronbach’s alpha coefficient, where a Cronbach alpha coefficient above 0.7 was considered acceptable.^10^ The QPC-COPS scale was further assessed for stability by correlating scores obtained on two occasions (2 weeks apart), by using the intra-class correlation coefficient (ICC). An ICC above 0.60 indicated satisfactory stability and above 0.80 is considered excellent.^10,33^

### Ethical Considerations

Ethical guidelines for medical research were followed regarding integrity, confidentiality, and the voluntariness of the participants.^34^ The participants were given oral and written information about the study, and all participants gave their written consent. Approval was given by the Norwegian Centre for Research Data (NSD) Ref: 54574.

## Results

### Translation and Cultural Adaption of the QPC-OPS Instrument to QPC-COPS

Only a few minor linguistic changes and concepts needed to be discussed before agreement was reached on the instrument Quality in the Psychiatric Care-Community outpatient staff (QPC-COPS). The need for only minor changes and the reason why the translation process was uncomplicated are explained by the fact that Norwegian and Swedish mental health care settings generally share a number of cultural and linguistic factors.

### Face and Content Validity of the QPC-COPS Instrument

Overall, the Norwegian mental health staff considered the items in the QPC-COPS to be important in addressing the quality in care. The items were also experienced as relevant to the Norwegian community mental health care culture. As a result of the expert panel discussions, there was further clarification of the perspective from which the mental health staff were to respond to the instrument items. It was made more explicit in the introductory part of the instrument that the mental health staff should respond to how they perceived the Quality in Care from their own perspective, and not from the patient perspective.

Based on the expert panels’ overall evaluation of the items, it was not necessary to add or delete any item, and the instrument was considered to have adequate face and content validity; thus, the subsequent test of its reliability (internal consistency and test–retest) was justified.

### Internal Consistency and Test–Retest Reliability

The Cronbach’s alpha (Table 2) demonstrated adequate internal consistency for the total QPC-COPS and all dimensions, except the next of kin dimension. The test–retest for the total QPC-COPS was considered excellent, demonstrating an ICC of 0.90 (95% CI 0.87:0.94). The test–retest of the eight dimensions was adequate, demonstrating ICCs in the range 0.58–0.74 (Table 2).

**Table 2:** Test–retest of QPC COPS dimensions

		Test			Retest			
	Dimensions	M	SD	α	M	SD	α	ICC
1	Encounter (6 items)	3.48	0.40	0.85	3.38	0.48	0.89	0.58
2	Participation-empowerment (3 items)	3.14	0.51	0.77	3.13	0.50	0.82	0.67
3	Participation-information (5 items)	3.15	0.45	0.86	3.07	0.54	0.72	0.68
4	Discharge (3 items)	3.27	0.42	0.68	3.19	0.48	0.51	0.71
5	Support (4 items)	2.95	0.46	0.80	3.28	0.52	0.68	0.71
6	Environment (3 items)	2.90	0.45	0.72	2.93	0.42	0.68	0.65
7	Next of kin (2 items)	2.99	0.60	0.42	3.10	0.56	0.33	0.58
8	Accessibility (4 items)	2.91	0.44	0.74	2.84	0.48	0.59	0.74

*N* = 64

α Cronbach’s alpha

ICC (intraclass correlations)

The total sum score of the QPC-COPS for the first test occasion of the test–retest demonstrated a mean score of 3.19 (Sd 0.32) (Table 3).

**Table 3:** Quality in Psychiatric Care-Outpatient Staff (QPC-COPS) among health professionals in community mental health services

QPC-COPS items by dimensions	M	SD
Total QPC-COPS (30 items)	3.19	0.32
1. Encounter (6 items)	3.48	0.40
11. Shows empathy	3.50	0.50
12. Cares if patients get angry	3.42	0.58
15. Respects the patients	3.57	0.52
18. Shows understanding	3.48	0.50
20. Has time to listen	3.29	0.58
25. Cares about patients’ care	3.65	0.47
2. Participation-empowerment (3 items)	3.14	0.51
1. Patients have influence over their care	3.23	0.55
5. Patients’ view of the right care is respected	2.98	0.65
6. Patients take part in decision-making about their care	3.20	0.59
3. Participation-information (5 items)	3.15	0.45
13. Benefit drawn from the patient’s earlier experience of treatment	3.34	0.56
14. Patients helped to recognize signs of deterioration	3.21	0.67
27. Patients informed in a way that they understand	3.06	0.63
29. Patients have knowledge about their mental troubles	3.28	0.62
30. Patients receive information about treatment alternatives	2.87	0.71
4. Discharge (3 items)	3.27	0.42
8. Patients’ treatment helps	2.93	0.55
17. Patients are offered help in finding occupation	3.20	0.69
21. Patients know where to turn	3.69	0.52
5. Support (4 items)	3.39	0.47
19. Stops the patients from hurting others	3.06	0.39
22. Stops the patients from hurting themselves	3.09	0.68
23. Nothing shameful about having mental troubles	3.59	0.60
24. Shame and guilt must not get in the way	3.64	0.60
6. Environment (3 items)	2.90	0.45
2. Trust the health care professionals	3.10	0.50
4. Feel secure in their own home	2.85	0.68
9. Feel secure in their own neighbourhood	2.75	0.56
7. Next of kin (2 items)	2.99	0.60
10. Next of kin invited to take part	2.54	0.85
28. Respects next of kin	3.65	0.47
8. Accessibility (4 items)	2.91	0.44
3. Easy for the patients to meet the contact person	3.17	0.65
7. Easy for the patients to get an appointment with the contact person	3.29	0.60
16. Easy for the patients to reach the clinic by phone	3.10	0.63
26. Easy for the patients to meet the doctor/GP	2.06	0.73

The staff’s rating on the occasion of the first test (Table 3) showed that they perceived the quality in care as generally high for the full QPC-COPS instrument. The highest ratings were found in the encounter dimension and the lowest in the environment dimension. The highest score on a single item was given on the item “Patients know where to turn” (discharge dimension) and the lowest score on the item “Easy for the patients to meet the doctor/GP” (accessibility dimension).

## Discussion

Since there is currently no psychometrically evaluated instrument to measure the quality of community mental health care in  Norwegian from staff perspectives, the aim of the study was to validate and culturally adapt the Swedish validated instrument QPC-OPS to the Norwegian community mental health service. The new Norwegian instrument was named QPC-COPS. The study further aimed to psychometrically evaluate the new QPC-COPS in a Norwegian sample of mental health staff. Based on the expert panel discussions, the results show that the QPC-COPS instrument has adequate face validity and excellent reliability (internal consistency and retest-reliability). The QPC-COPS thus provides the community mental health services with a validated and reliable instrument that meets the Norwegian central government’s call to improve mental health services to the population and to strengthen coordination and cooperation between the community mental health service and the specialist mental health services.^35^

The results also demonstrate the value of validating and culturally adapting an already existing and well-established psychometrically evaluated instrument from a comparable mental health care context rather than developing a completely new one and suggest that this solution should be prioritized. To ensure cultural equivalence and thus maintain the content validity across different cultures, the instrument was carefully translated and adapted to the Norwegian culture and community mental health setting. The minor challenges during translation and the cultural adaptation of QPC-COPS concerned semantic issues and were to be expected since Norway and Sweden differ somewhat in the organization of mental health services and care cultures. Despite the minor differences, a few linguistic changes were necessary before the adaptation of QPC-COPS was agreed upon by the expert panel. Thus, the expert interviews demonstrated that the adapted version was accurate, expressed the intended meanings of the items, and that each item was perceived as relevant to the Norwegian community mental health context.

Possibly due to the rigorous adaptation process, the QCP-COPS showed excellent reliability^33^, both regarding internal consistency and test–retest reliability. This is considered a core issue when choosing an instrument to assess or improve quality in health services. However, the next of kin dimension showed an alpha value below a satisfactory level. This can be understood as being a result of the low number of items (2 items) measuring this dimension. The addition of one or two items would probably increase its internal consistency.

Even though the main objective of this study was to describe the adaptation process and evaluate the QPC-COPS’ content validity and reliability, it is worth noting that the mental health staff in the present study perceived the quality in care in general as high. The highest rating was found in the encounter dimension, which is in line with previous studies in mental health services^36–39^ as well as in other health care contexts.^40^ This can be seen as a positive result for the community mental health service since encounters are an essential factor with regard to interpersonal relationships and quality in care.^6^

According to a previous study in community mental health using the QPC-COPS^39^, the mental health staff, as in our study, rated quality in the Environment dimension as lowest. This finding may indicate that the staff, relatively speaking, did not perceive the environment as safe for the patients. One possible explanation may be that mental health staff assessed patients’ living conditions as unacceptable, and this will result in moral and ethical issues for the professionals. The findings, however, may indicate that the environment is an area for improvement in comparison with other quality in care dimensions.

Some limitations of the study should be noted. Although the expert panel indicated face validity, more stringent tests of validity, such as construct validity evaluations using statistical methods (e.g., factor analysis) were not possible due to too few respondents. The general rule of thumb suggests a minimum of 150 respondents. Only 64 staff members agreed to participate though staff were recruited from 18 municipalities. Difficulties finding participants in questionnaire-based studies are, however, a general problem.^41^ Including more municipalities might have been a way to improve the number of respondents. Another limitation was that most of the participants were female nurses. On the other hand, this is representative of staffing within community mental health services.

## Conclusion

The adequacy of content and face validity, excellent internal consistency, and test–retest reliability findings in this study demonstrate that the Norwegian QPC-COPS is a valid and reliable instrument for measuring perception care quality in community mental health services. Additionally, the Norwegian QPC-COPS is an easily administered tool for use in quality monitoring and quality development. The instrument is considered ready for clinical use.

## Implications for Behavioral Health

To improve behavioral health services, the QPC-COPS provides an opportunity to monitor quality in care from the perspective of mental health staff. This might help to identify areas and dimensions for quality improvements in community mental health services in general. The 30-item QPC-COPS is an easy, time-efficient instrument that may support staff and leaders in identifying core behavioral health issues within mental health services areas for quality improvements.

### Recommendations for Further Research

Further research concerning translation and adaption of the QPC-COPS instrument for the community mental health services should address patients and next of kin. The research team plan to address these issues in forthcoming studies.
